# Associations between Various Inflammatory Markers and Carotid Findings in a Voluntary Asymptomatic Population Sample

**DOI:** 10.3390/ijms25179656

**Published:** 2024-09-06

**Authors:** Balázs Bence Nyárády, Edit Dósa, László Kőhidai, Éva Pállinger, Renáta Gubán, Ádám Szőnyi, Loretta Zsuzsa Kiss, Zsolt Bagyura

**Affiliations:** 1 Heart and Vascular Center, Semmelweis University, 1122 Budapest, Hungary; nyarady.balazs.bence@semmelweis.hu (B.B.N.); guban.renata@stud.semmelweis.hu (R.G.); szonyi.adam@stud.semmelweis.hu (Á.S.); bagyura.zsolt@semmelweis.hu (Z.B.); 2 Department of Genetics, Cell and Immunobiology, Semmelweis University, 1089 Budapest, Hungary; kohidai.laszlo@semmelweis.hu (L.K.); pallinger.eva@semmelweis.hu (É.P.); 3 Institute for Clinical Data Management, Semmelweis University, 1085 Budapest, Hungary

**Keywords:** inflammation, cytokines, chemokines, immunity, carotid intima-media thickness, atherosclerosis, cardiovascular risk factors

## Abstract

Cardiovascular disease (CVD) is the leading cause of morbidity and mortality worldwide, and atherosclerosis is the key factor promoting its development. Carotid intima-media thickening and the presence of carotid plaques are important indices of cardiovascular risk. In addition, inflammation is a major and complex factor in the development of atherosclerosis. The relationships between carotid atherosclerosis and certain inflammatory markers have rarely been studied in healthy individuals. Therefore, we aimed to investigate the associations between subclinical carotid atherosclerosis and various inflammatory biomarkers in a large Caucasian population free of evident CVD. In addition to recording study participants’ demographic characteristics, anthropometric characteristics, and atherosclerotic risk factors, laboratory tests were performed to measure levels of hemoglobin A1c (HbA1c), high-sensitivity C-reactive protein, and inflammatory cytokines/chemokines, including interleukin (IL)-1β, IL-6, IL-8, IL-10, IL-12p70, IL-17A, IL-18, IL-23, IL-33, interferon (IFN)-α2, IFN-γ, tumor necrosis factor-α, and monocyte chemoattractant protein (MCP)-1. This study included 264 asymptomatic individuals with a median age of 61.7 years (interquartile range, 54.5–67.5 years); 45.7% of participants were male. Participants were divided into two groups according to their carotid status: the normal carotid group, comprising 120 participants; and the pathological carotid group, comprising 144 participants. Compared with the normal carotid group, hypertension and diabetes mellitus were significantly more common and serum levels of HbA1c, IL-8, and MCP-1 were significantly higher in the pathological carotid group. Multivariate regression analysis revealed significant positive associations between pathological carotid findings and serum levels of IL-8 (highest tertile, OR: 2.4, *p* = 0.030) and MCP-1 (highest tertile, OR: 2.4, *p* = 0.040). Our results suggest that IL-8 and MCP-1 may serve as early indicators of subclinical atherosclerosis, thereby helping to identify individuals at increased risk of CVD before the onset of clinical symptoms.

## 1. Introduction

Cardiovascular disease (CVD) resulting from the complications of atherosclerosis is the leading cause of morbidity and mortality worldwide [1]. Atherosclerosis is a chronic inflammatory disease that is characterized by the accumulation of fatty deposits and pathologically activated inflammatory cells (e.g., macrophages and foam cells) in the inner layer of the arteries. The development of atherosclerosis is influenced by a number of factors, including genetic factors, lifestyle factors, and systemic diseases such as diabetes mellitus, but inflammation also plays a fundamental role [2]. Although CVD symptoms are associated with intimal thickening, it is increasingly being recognized that the composition of plaques determines an individual’s risk of cardiovascular complications [3,4,5].

**Figure 1:**
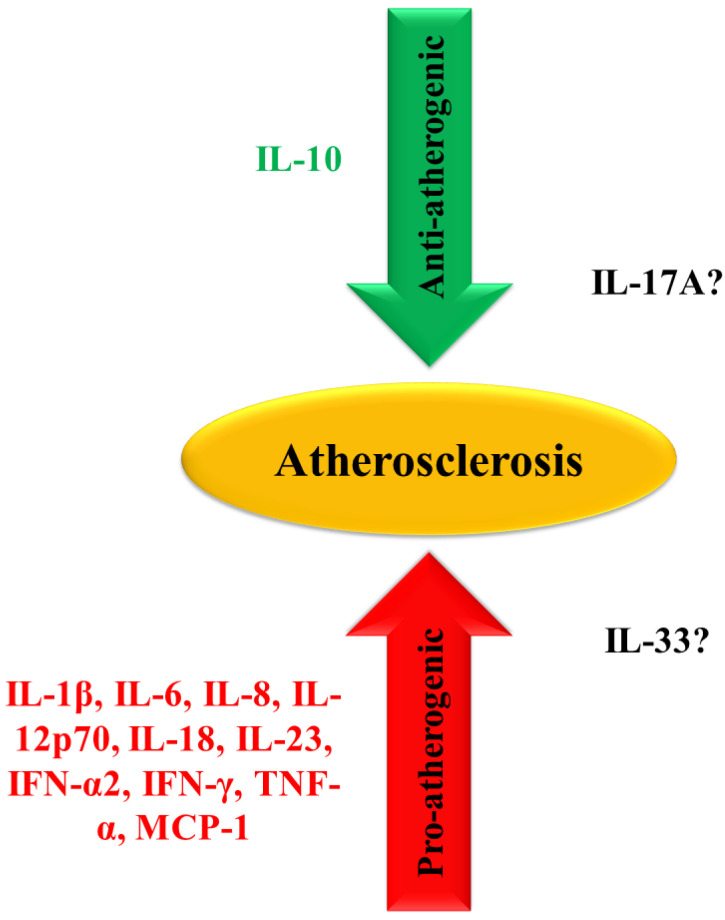
Pro- and anti-atherogenic cytokines. *IFN*, interferon; *IL*, interleukin; *IL-12p70*, heterodimer of IL-12 composed of disulfide-bonded p35 and p40 subunits; *MCP-1*, monocyte chemoattractant protein-1; *TNF-α*, tumor necrosis factor-α.

Cytokines are key regulators of inflammation that function through a complex and sometimes contradictory network of interactions. Based on data from experiments on transgenic animals, cytokines can be classified as either pro- or anti-atherogenic (Figure 1). Overexpression of pro-atherogenic cytokines—including interleukin (IL)-1β, IL-6, IL-8, IL-12p70, IL-18, IL-23, interferons (IFNs), tumor necrosis factor (TNF)-α, and monocyte chemoattractant protein (MCP)-1—promotes vascular inflammation and atherosclerosis, whereas their mutation reduces both the number and size of atherosclerotic lesions. In contrast, anti-inflammatory cytokines such as IL-5, IL-10, IL-19, IL-35, and transforming growth factor (TGF)-β inhibit vascular inflammation and atherosclerosis. Deficiencies in these anti-inflammatory cytokines increase both the number and size of atherosclerotic lesions. Although cytokines have emerged as promising new targets for the treatment of atherosclerosis, several controversial results have been reported, mainly due to the local cytokine milieu interactions [6]. As the local cytokine milieu determines the ultimate effects of individual cytokines, it is advisable to consider cytokines in the context of other specific markers when assessing their potential applicability as biomarkers.

Serum levels of high-sensitivity C-reactive protein (hsCRP) have been shown to be elevated in individuals decades before they first experience an acute ischemic event. C-reactive protein production results from the downstream signaling of IL-6 and, to a lesser extent, IL-1β, and the roles of these cytokines in predicting future vascular risk have been investigated in several cohorts [7]. Interleukin-1 is a central proinflammatory mediator that can induce leukocyte adhesion to endothelial cells, lead to procoagulant activity, and serve as a mitogen for vascular smooth muscle cells (VSMCs) [8]. In mouse knockout models, IL-1β deficiency is associated with reduced atherosclerotic lesion formation [9]. Furthermore, high serum levels of IL-6 correlate with endothelial dysfunction, arterial stiffness, and atherosclerosis severity [10].

Interleukin-10 is a predominantly anti-inflammatory cytokine that is known to suppress and resolve inflammatory responses, thereby maintaining immune homeostasis [11]. Some studies suggest that IL-10 may have a preventive effect against the development and progression of atherosclerosis, as higher serum levels of IL-10 are associated with lower carotid intima-media thickness (CIMT) values [12,13]. In addition, a positive correlation was observed between CIMT and serum levels of IL-10 in patients with a human immunodeficiency virus (HIV) infection [14].

Despite being actively studied, the roles of IL-17 and IL-33 in atherosclerosis are currently unclear [15,16,17].

Interferons belong to a cytokine family that plays a complex role in atherosclerosis; the best characterized member of this family is IFN-γ. Activated cellular components of atherosclerotic lesions (macrophages, T cells, and VSMCs) produce IFN-γ, which regulates the recruitment and behavior of immune cells. For example, IFN-γ polarizes macrophages toward the proinflammatory M1 phenotype, which results in the progression and enlargement of atherosclerotic plaques through the production of a fibrous cap. Vascular smooth muscle cells play an important role in the stabilization of atherosclerotic plaques [18]. In contrast, INF-γ inhibits the production of collagens I and III by VSMCs, thereby increasing the vulnerability of the plaques [19]. Type I IFNs are also secreted by macrophages. Animal studies have demonstrated that IFN-α and IFN-β—which are both type I IFNs—increase the sizes of atherosclerotic lesions [20,21].

Tumor necrosis factor-α is a multifunctional mediator of atherosclerosis that functions through the induction of endothelial adhesion molecules and the subsequent recruitment of monocytes to the intima, followed by the downregulation of endothelial nitric oxide synthase and the transformation of endothelial cells into fibroblasts [22,23]. Tumor necrosis factor-α has also been reported to play a role in lipid metabolism, including the suppression of key enzymes such as 7α-hydroxylase and lipoprotein lipase, thereby stimulating triglyceride production in the liver [24].

Macrophages play an important role in all stages of atherosclerotic plaque formation. Circulating monocytes are recruited to the site of atherosclerotic lesions, where they differentiate into macrophages [25]. Experiments with transgenic mice have shown that the recruitment of monocytes is tightly regulated by the interaction of the MCP-1 with CC motif chemokine receptor (CCR) 2. Importantly, mice that are deficient in MCP-1 or its receptor (CCR2) have significantly fewer atherosclerotic lesions compared with other mice [26]. Monocyte chemoattractant protein-1, also known as chemokine CC-motif ligand (CCL) 2, belongs to the family of CC chemokines. Although endothelial cells are the primary source of MCP-1, they are not its only source. Activated endothelial cells, such as those induced by oxidative stress, overexpress MCP-1, which controls monocytes’ migration and infiltration into the arterial wall [27]. Higher serum levels of MCP-1 are associated with increased recruitment of monocytes into the arterial wall, which in turn promotes inflammation and plaque formation [28].

Carotid intima-media thickness, which is an important indicator of subclinical atherosclerosis, has become an established surrogate marker for CVD; it provides a non-invasive, radiation-free, easy-to-perform method to assess arterial wall thickening and provides essential information about the early stages of atherosclerosis [29]. To the best of our knowledge, no previous study has investigated the associations between CIMT values and those of a broad panel of circulating inflammatory biomarkers in a healthy population. Previous studies did not examine healthy populations but rather examined groups with chronic immunological conditions (HIV infection, autoimmune diseases, etc.) in which the serum levels of these biomarkers are usually abnormal. By studying these relationships in a large Caucasian population that is free of evident CVD, we can better understand the complex interactions between inflammatory mediators and changes in the arterial wall, which may provide new insights into the inflammatory pathways that precipitate atherosclerosis. In the present study, we demonstrate the importance of circulating proinflammatory mediators in the pathogenesis of CVD.

## 2. Results

**Figure 2:**
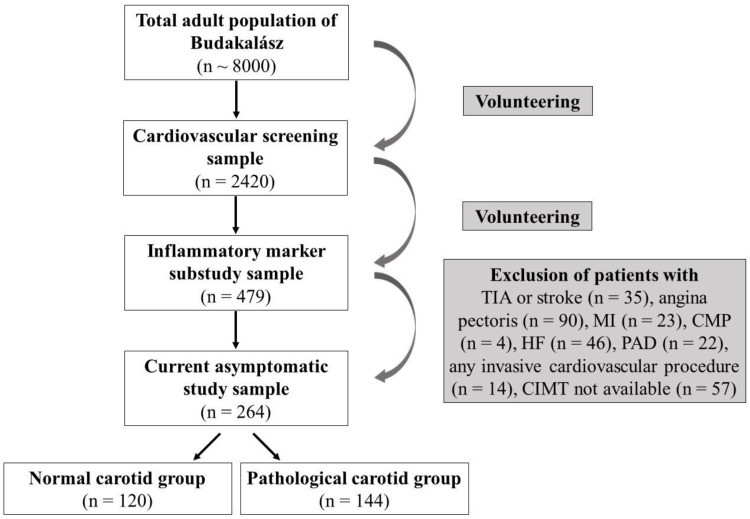
Study flowchart. *CIMT*, carotid intima-media thickness; *CMP*, cardiomyopathy; *HF*, heart failure; *MI*, myocardial infarction; *PAD*, peripheral arterial disease; *TIA*, transient ischemic attack.

Inflammatory markers were measured in 479 participants. Subjects with a history of TIA or stroke (n = 35 (7.3%)), angina pectoris (n = 90 (19%)), MI (n = 23 (4.8%)), CMP (n = 4 (0.8%)), HF (n = 46 (9.6%)), or symptomatic PAD (n = 22 (4.6%)), or who had undergone any invasive procedure (n = 14 (2.9%)) were excluded from further analysis. Due to technical limitations, CIMT measurements could not be taken in 57 (11.9%) participants. Thus, our study population consisted of 264 asymptomatic Caucasian individuals (median age, 61.7 years (IQR, 54.5–67.5 years)), 45.7% of whom were male (Figure 2).

Participants were divided into two groups according to their carotid status: the normal carotid group, consisting of 120 (45.5%) participants, and the pathological carotid group, consisting of 144 (54.5%) participants. In the pathological carotid group, no stenosis causing significant hemodynamic changes was detected in any of the participants. Sixteen (11.1%) participants had more than one plaque. The total plaque area was 14.9 mm^2^ (IQR, 8.5–26.4 mm^2^) on the left side and 19.1 mm^2^ (IQR, 9.7–25.1 mm^2^) on the right side. Eighty-one percent of the plaques were sclerotic or calcified. None of the participants had exulcerated and/or hypoechogenic (vulnerable) plaques. Table 1 shows the demographic data and cardiovascular risk factors of the participants in the two groups. Participants had a median body mass index (BMI) of 27.7 kg/m^2^ (IQR, 25.1–31.0 kg/m^2^), a median HbA1c level of 5.7% (IQR, 5.4–6.0%), and a median hsCRP level of 1.6 mg/L (IQR, 0.8–3.5 mg/L). Of all participants, 30 (11.3%) were smokers, 143 (54.0%) had hypertension, 111 (41.9%) had hyperlipidemia, and 33 (12.5%) had diabetes mellitus. There were significant differences between the normal and pathological carotid groups in terms of age, history of hypertension, diabetes mellitus, and HbA1c levels, as participants in the pathological carotid group had higher numbers of comorbidities compared with participants in the normal carotid group.

The median serum levels of inflammatory markers in the two study groups are presented in Table 2. Among all of these markers, only the serum levels of IL-8 and MCP-1 showed a significant difference between the two groups.

The prevalences of pathological carotid findings in participants divided into IL-8 tertiles were 37.6% in the first tertile, 46.1% in the second tertile, and 55.5% in the third tertile (*p* = 0.047). The prevalences of pathological carotid findings in participants divided into MCP-1 tertiles were 31.4% in the first tertile, 47.7% in the second tertile, and 57.8% in the third tertile (*p* = 0.002).

### 2.1. Logistic Regression Analyses: Univariate Models

Logistic regression analysis was performed with the presence of pathological carotid findings as the dependent variable, including IL-8- and MCP-1-tertile variables as independent variables and using the first tertile as the reference category. The results show that patients with serum levels of IL-8 (OR: 2.3, *p* = 0.040) or MCP-1 in the third tertile (OR: 2.4, *p* = 0.034) were more likely to have pathological carotid findings compared with participants with serum levels of IL-8 and MCP-1 in the first tertile.

### 2.2. Logistic Regression Analyses: Multivariate Models

In a multivariate logistic regression model adjusted for age, sex, BMI, smoking, hypertension, hyperlipidemia, diabetes mellitus, HbA1c, creatinine, and hsCRP, the third tertiles of IL-8 and MCP-1 showed significant positive associations (OR: 2.4, *p* = 0.030 and OR: 2.4, *p* = 0.040, respectively) with the presence of pathological carotid findings compared with the first tertiles of IL-8 and MCP-1 as the reference categories.

## 3. Discussion

In this study, we aimed to investigate the relationships between subclinical carotid atherosclerosis and various inflammatory biomarkers in a large Caucasian population without evident CVD. Our study is unique in that it thoroughly examined a wide range of biomarkers reflecting different facets of the inflammatory response, including IL-1β, IL-6, IL-10, IL-12p70, IL-17A, IL-18, IL-23, IL-33, IFN-α2, IFN-γ, TNF-α, and MCP-1. We identified correlations between carotid atherosclerosis and elevated serum levels of IL-8 and MCP-1, especially among participants with levels of IL-8 and MCP-1 in the highest tertiles (OR: 2.4, *p* = 0.030 and OR: 2.4, *p* = 0.040, respectively); this reveals possible roles for these inflammatory mediators in the early stages of atherosclerosis development. Previous studies have investigated IL-8 and MCP-1 only in certain patient groups such as HIV-infected patients, autoimmune patients (with psoriasis, rheumatoid arthritis, or type 1 diabetes mellitus), or in patients with immune-mediated conditions. However, the results from these studies, including conclusions on the predictive value of the biomarkers tested, cannot be generalized to healthy populations. Therefore, the aim of our study was to examine the possible relationships between pathological carotid findings and proinflammatory cytokines in a population of healthy subjects with a broad age range.

A large European cohort (the IMPROVE study) investigated the causal link between CIMT and IL-8 and confirmed the role of IL-8 in promoting subclinical atherosclerosis in different patient populations [30]. Tuncay et al. [31] found significantly elevated serum levels of IL-8 in children with chronic kidney disease, but no significant correlation was found between CIMT values and serum IL-8 levels. In a clinical study analyzing the effect of atorvastatin treatment on CIMT changes in patients with stable coronary artery disease, not only a regression of CIMT but also a parallel decrease in serum IL-8 levels was observed [32].

As mentioned earlier, MCP-1 promotes vascular inflammation, which can lead to increased CIMT and atherosclerotic plaque formation. Increased values of CIMT indicate the presence of more advanced subclinical atherosclerosis [28]. In other studies investigating healthy populations, MCP-1 has consistently been reported to be a significant biomarker for CIMT. However, these studies have focused on the following specific populations: young Black women, postmenopausal women without CVD, and individuals at increased cardiovascular risk [28,33]. In healthy young children and children with untreated primary hypertension, MCP-1 showed no significant relationship with vascular characteristics, suggesting that its effect may be more pronounced in older, at-risk populations [34,35]. Studies examining serum MCP-1 levels in HIV-infected individuals who were in a stable state while receiving antiretroviral therapy showed a significant positive correlation between MCP-1 levels and CIMT values and their changes [36,37,38]. Studies in patients with autoimmune diseases (e.g., psoriasis and rheumatoid arthritis), as well as in patients with type 1 diabetes mellitus and autosomal dominant polycystic kidney disease, suggest that MCP-1 may play a role in promoting vascular inflammation and atherosclerosis in these patients [39,40,41,42,43,44].

The relationship between CIMT values and combined serum levels of IL-8 and MCP-1 has rarely been investigated. Hassan et al. [45] studied a South African population of chronic kidney disease patients and revealed strong associations between increased CIMT and higher serum levels of IL-8 and MCP-1. We noted similar correlations but in a healthy Caucasian cohort, which has not been described previously.

Our results suggest that systemic inflammation, as indicated by high serum levels of IL-8 and MCP-1, may have significant effects on carotid artery status and on early atherosclerotic changes, even in the absence of evident CVD. These findings highlight the importance of considering a wider range of inflammatory mediators when assessing cardiovascular risk. Our results also underline the need to further investigate the complex relationships between different inflammatory mediators and alterations in the arterial wall. By understanding these relationships, targeted anti-inflammatory therapies could be developed to prevent the progression of atherosclerosis.

### Limitations

Our single-center study has several limitations. Our study sample may not be representative as it was based on a voluntary screening process, which may have resulted in higher-risk but more health-conscious people being more likely to participate in this study than healthy people were. The cross-sectional design of this study limited the ability to establish cause-and-effect relationships between CIMT changes and serum levels of biomarkers. Longitudinal studies are needed to confirm these associations and to determine whether changes in the serum levels of biomarkers precede the development of atherosclerosis.

## 4. Patients and Methods

The present study was part of the 2011–2013 Budakalász Health Survey, which is a cross-sectional voluntary (cardio)vascular screening program for adults over 20 years of age in Budakalász [46]. The project was approved by the Scientific and Ethical Committee of the Medical Research Council (approval number 8224-0/2011/ECU (265/PI/11)). All procedures were conducted in accordance with the 1975 Declaration of Helsinki, which was updated in 2000, and the ethical guidelines for human experimentation of the relevant national and institutional councils [47]. Written informed consent was obtained from all study participants.

In addition to collecting demographic (age and sex) and anthropometric (weight and height) data, laboratory tests were performed. The medical histories obtained for participants focused on the presence and management of atherosclerotic risk factors—such as smoking, hypertension, and diabetes mellitus—and of risk factors for (cardio)vascular diseases, including transient ischemic attack (TIA), stroke, angina pectoris, myocardial infarction (MI), cardiomyopathy (CMP), heart failure (HF), and peripheral arterial disease (PAD). Non-fasting venous blood samples were taken on site and analyzed in our laboratory. Laboratory assays were performed to measure levels of high-density lipoprotein cholesterol, low-density lipoprotein cholesterol (LDL-C), hsCRP, hemoglobin A1c (HbA1c), and inflammatory cytokines/chemokines, including IL-1β, IL-6, IL-8, IL-10, IL-12p70, IL-17A, IL-18, IL-23, IL-33, IFN-α2, IFN-γ, TNF-α, and MCP-1.

The definitions and methods used in this study were described in detail in our previous publication [48]. If a participant smoked one or more cigarettes a day, then he or she was considered an active smoker. If the participant had hypertension or diabetes mellitus, or if these conditions were documented in previous medical records, then the participant was considered to have these comorbidities. Hyperlipidemia was defined as an elevated (3.4 mmol/L or higher) LDL-C level measured at the time of screening. All laboratory tests were performed in our central laboratory under strict quality-control processes. Hemoglobin A1c and hsCRP levels were measured by immunoturbidimetry (Beckman Coulter DxC 700 AU; Beckman Coulter Inc., Brea, CA, USA). The following inflammatory cytokines/chemokines were measured by flow cytometry-based multiplex immunoassay (LEGENDplex Human Inflammation Panel 1 (13-plex); BioLegend, San Diego, CA, USA) according to the manufacturer’s instructions: IL-1β, IL-6, IL-8, IL-10, IL-12p70, IL-17A, IL-18, IL-23, IL-33, IFN-α2, IFN-γ, TNF-α, and MCP-1.

At end diastole, high-quality longitudinal B-mode ultrasound images (VIVID I; GE HealthCare Inc., Wauwatosa, WI, USA) of both common carotid arteries (CCAs) were obtained by the same person for each participant. For the CIMT assessment, a 10 mm straight arterial segment that was free of plaques was marked on the far wall of the CCA at least 5 mm below the bifurcation, according to the principles of the 2012 Mannheim Consensus [33]. Then, the semi-automated software EchoPAC BT11 (GE HealthCare Inc., Wauwatosa, WI, USA) calculated and recorded the maximum and mean CIMT values on both sides. A CIMT value of 0.9 mm or greater was considered abnormal. When present, carotid plaques were evaluated for size and morphology. Two groups were distinguished by these results: a normal carotid group, consisting of participants with a CIMT value below 0.9 mm and no visible carotid plaques; and a pathological carotid group, consisting of participants with a CIMT value of 0.9 mm or greater, with or without the presence of plaques.

### Statistical Analysis

Statistical analyses were performed using both Excel and SPSS for Windows version 25.0 (IBM, Armonk, NY, USA). Due to the distributions of the variables, continuous variables were presented as median and interquartile range (IQR) and the Mann–Whitney U-test was used for comparisons. Categorical variables were presented as numerical and percentage values and compared using the Chi-square test. Among the inflammatory cytokine/chemokine variables, those that showed a significant correlation with CIMT (IL-8 and MCP-1) were used to create categorical variables describing tertiles; these categorical variables were included in a regression model. The cut-off values for the IL-8 tertiles used in the analysis were as follows: first tertile, values below 23.69 pg/mL; second tertile, values between 23.69 pg/mL and 26.24 pg/mL; and third tertile, values above 26.24 pg/mL. Monocyte chemoattractant protein-1 tertiles were calculated as follows: first tertile, values below 86.51 pg/mL; second tertile, values between 86.51 pg/mL and 117.27 pg/mL; and third tertile, values above 117.27 pg/mL. Logistic regression analysis adjusting for age, sex, and atherosclerosis risk factors was performed to examine the association between CIMT and the aforementioned inflammatory markers in a multivariate model. All analyzes were two-sided, and *p* < 0.05 was considered significant.

## Figures and Tables

**Table 1:** Participants’ demographics, cardiovascular risk factors, hemoglobin A1c levels, creatinine levels, and high-sensitivity C-reactive protein levels.

Parameters	All Participants (n = 264)	Normal Carotid Group (n = 120)	Pathological Carotid Group (n = 144)	*p*-Value
Age (years), median (IQR)	61.7 (54.4–67.5)	56.7 (46.3–62.7)	66.0 (61.8–71.3)	<0.001
Sex (male), n (%)	121 (45.7)	63 (44.1)	58 (47.5)	NS
BMI (kg/m^2^), median (IQR)	27.7 (25.1–31.0)	27.5 (24.3–30.8)	28.2 (25.3–31.3)	NS
Smoking, n (%)	30 (11.3)	18 (12.6)	12 (9.8)	NS
HT, n (%)	143 (54.0)	60 (42.0)	83 (68.0)	<0.001
HLP, n (%)	111 (41.9)	70 (52.1%)	57 (49.1%)	NS
DM, n (%)	33 (12.5)	8 (5.6)	25 (20.5)	<0.001
HbA1c (%), median (IQR)	5.7 (5.4–6.0)	5.6 (5.4–5.9)	5.8 (5.4–6.2)	0.015
Creatinine (mmol/L), median (IQR)	77.0 (66.0–88.5)	75.0 (64.0–86.0)	80.0 (66.8–91.0)	NS
hsCRP (mg/L), median (IQR)	1.6 (0.8–3.5)	1.5 (0.7–4.3)	1.7 (1.0–3.0)	NS

*BMI*, body mass index; *DM*, diabetes mellitus; *HbA1c*, hemoglobin A1c; *HLP*, hyperlipidemia; *hsCRP*, high-sensitivity C-reactive protein; *HT*, hypertension; *IQR*, interquartile range; *NS*, non-significant.

**Table 2:** Participants’ serum levels of inflammatory markers based on carotid artery status.

Inflammatory Markers, Median (IQR)	All Participants (n = 264)	Normal Carotid Group (n = 120)	Pathological Carotid Group (n = 144)	*p*-Value
IL-1β	56.5 (56.1–56.9)	56.5 (56.2–56.9)	56.4 (56.0–56.9)	NS
IL-6	32.4 (30.9–34.7)	32.4 (31.0–34.1)	32.5 (30.7–34.9)	NS
IL-8	25.0 (23.0–26.9)	24.8 (22.7–26.5)	25.4 (23.6–27.4)	0.024
IL-10	25.6 (25.1–26.1)	25.6 (25.1–26.0)	25.6 (25.0–26.2)	NS
IL-12p70	6.1 (5.3–7.1)	6.1 (5.3–7.1)	6.1 (5.1–7.2)	NS
IL-17A	76.1 (74.5–77.4)	76.1 (74.8–77.4)	76 (74.1–77.5)	NS
IL-18	90.0 (58.6–133.6)	88.1 (58.9–131.6)	92.9 (58.3–144)	NS
IL-23	49.9 (22.6–61.7)	49.9 (24.8–61.7)	50.5 (16.9–61.4)	NS
IL-33	645.9 (601.9–666.7)	644.4 (601.0–665.4)	647.5 (602.4–668.0)	NS
IFN-α2	40.3 (40.0–41.8)	40.4 (40.0–42.0)	40.3 (39.9–41.6)	NS
IFN-γ	28.1 (26.0–31.0)	27.9 (26.2–31.6)	28.2 (25.6–30.2)	NS
TNF-α	14.8 (12.6–18.9)	15.0 (12.7–18.8)	14.7 (12.3–19.2)	NS
MCP-1	102.5 (79.9–125.6)	91.6 (74.1–118.3)	110.8 (88.0–134.4)	<0.001

*IFN*, interferon; *IL*, interleukin; *IL-12p70*, heterodimer of IL-12 composed of disulfide-bonded p35 and p40 subunits; *IQR*, interquartile range; *MCP-1*, monocyte chemoattractant protein-1; *NS*, non-significant; *TNF-α*, tumor necrosis factor-α.

## Data Availability

The data presented in this study are available on request from the corresponding author. The data are not publicly available due to reasons pertaining to patient privacy.
